# Zastaprazan, a Novel Potassium-Competitive Acid Blocker, for Acid-Related Disorders

**DOI:** 10.3390/jcm15103700

**Published:** 2026-05-11

**Authors:** Gwang Ha Kim, Dong Chan Joo, Moon Won Lee, Bong Eun Lee

**Affiliations:** 1 Department of Internal Medicine, Pusan National University School of Medicine, Busan 49241, Republic of Korea; asclllepios@gmail.com (D.C.J.); neofaceoff@hanmail.net (M.W.L.); bongsul@hanmail.net (B.E.L.); 2 Biomedical Research Institute, Pusan National University Hospital, Busan 49241, Republic of Korea

**Keywords:** zastaprazan, potassium-competitive acid blockers, gastroesophageal reflux disease, gastric ulcer, acid-related disorders

## Abstract

Proton pump inhibitors (PPIs) serve as the primary treatment for acid-related disorders, such as gastroesophageal reflux disease and peptic ulcer disease. Although PPIs are regarded as the first-line medication for acid suppression, they have notable limitations, including the need for acid-mediated activation, a short half-life and duration of action, and metabolic variability. Zastaprazan is a newly developed potassium-competitive acid blocker (P-CAB) that competitively and reversibly inhibits acid production and secretion. Like other P-CABs, zastaprazan exhibits pharmacodynamic and pharmacokinetic properties that differs significantly from those of PPIs. These differences offer potential advantages, such as rapid, robust, and long-standing acid suppression, the lack of CYP2C19 metabolism, and no need for conversion into an active form. Completed clinical trials of zastaprazan have demonstrated comparable or superior efficacy to that of PPIs for the healing of erosive esophagitis and gastric ulcers without concerning safety signals. Notably, in erosive esophagitis, zastaprazan 20 mg demonstrated faster healing at week 4 compared to esomeprazole 40 mg, whereas in gastric ulcers, zastaprazan 20 mg achieved a 100% healing rate at 8 weeks compared to 97.1% with lansoprazole 30 mg. Zastaprazan is approved in the Republic of Korea for the treatment of erosive esophagitis and gastric ulcer and is undergoing phase III clinical trials for the prevention of non-steroidal anti-inflammatory drug-induced peptic ulcer and the treatment of non-erosive reflux disease. In this review, we summarize and discuss the pharmacology, efficacy, and safety of zastaprazan in acid-related disorders.

## 1. Introduction

Acid-related disorders such as gastroesophageal reflux disease (GERD) and peptic ulcer disease (PUD), are among the most common gastroenterological conditions encountered in clinical practice [1]. A recent study reported that the global prevalence of GERD is 13.3%, with regional variations such as 10.0% in Asia, 15.4% in North America, and 17.1% in Europe [2]. PUD reached a global prevalence of approximately 8.1 million in 2019, marking a 25.8% increase compared to 1990 [3]. The classic symptoms of heartburn and regurgitation in GERD, as well as epigastric pain in PUD, have a significant negative impact on the quality of life for those affected. These conditions are associated with serious complications, including erosive esophagitis, esophageal strictures, Barrett’s esophagus, gastrointestinal bleeding, and perforation.

Proton pump inhibitors (PPIs) are a mainstay treatment for acid-related disorders, supported by well-established data that demonstrate their superior efficacy in healing and symptom relief compared to placebo, antacids, and histamine-2-receptor antagonists (H_2_RAs) [4,5,6]. Many studies have demonstrated that maintaining a gastric pH > 4 for prolonged periods enhances ulcer healing rates and mucosal recovery in acid-related disorders [7]. Even though they have been shown to be superior to other commonly available acid suppression medications, PPIs have significant limitations, such as their status as prodrugs that require acid-mediated activation in the secretory canaliculus, their relatively slow onset of action (taking 3 to 5 days to reach maximum efficacy), their short plasma half-life and duration of action resulting in insufficient suppression of nocturnal acid secretion, and their metabolic variability influenced by cytochrome P450 (CYP) 2C19 polymorphism [8,9]. These limitations may affect the effectiveness of PPIs, and up to 40% of patients treated with PPIs for GERD report persistent symptoms while taking these medications as recommended [1]. Furthermore, about 10 to 20% of patients with Los Angeles (LA) grade C and D esophagitis do not experience complete healing even after 8 weeks of double-dose PPI therapy [10], and 15% to 30% of patients with acid-related disorders continue to experience symptoms despite PPI treatment [11].

To address these unmet needs, a novel class of drugs, potassium-competitive acid blockers (P-CABs), has been developed. Compared to PPIs, P-CABs provide a faster and more sustained inhibition of proton pumps and gastric acid secretion [12,13]. Several P-CABs, including vonoprazan, tegoprazan, and fexuprazan, have gained regulatory approval and are now available in various regions worldwide. P-CABs are molecules that, after systemic absorption, accumulate in the secretory canaliculus of the parietal cell, where they bind ionically to the H^+^, K^+^-ATPase, inhibiting acid formation and secretion in a reversible and competitive manner. Mechanistically, the substantial size of P-CABs prevents K^+^ from binding to the proton pump, thereby inhibiting the exchange of H^+^ and K^+^ ions necessary for the acid secretion process [14]. Because they are acid-stable and administered as active drugs rather than prodrugs, P-CABs do not require enteric coating, reach peak plasma levels shortly after administration, can be taken regardless of food intake in most cases, and provide an immediate effect from the first dose. They also have a longer plasma half-life and duration of action than PPIs, allowing for effective control of nocturnal acid secretion. Finally, most P-CABs are not metabolized by CYP2C19, minimizing efficacy variability due to genetic differences and resulting in more consistent pharmacodynamic effects across the patient population.

Zastaprazan, a new P-CAB developed by Onconic Therapeutics (Seoul, Republic of Korea) and marketed as JAQBO^®^ (Jeil Pharmaceutical Co. Ltd., Seoul, Republic of Korea), received its first regulatory approval in the Republic of Korea in April 2024, for the treatment of erosive GERD [15], and its indication was later expanded to include the treatment of gastric ulcer in June 2025. In this narrative review, we provide a summary of the current evidence concerning the development, pharmacology, efficacy, and safety of zastaprazan across acid-related disorders including erosive esophagitis and gastric ulcers.

## 2. Pharmacokinetics and Pharmacodynamics

Preclinical studies have demonstrated that zastaprazan is a highly effective and selective inhibitor of gastric H^+^/K^+^-ATPase activity, with a half-maximal inhibitory concentration of 16.7 nM and more than 600-fold selectivity over Na^+^/K^+^-ATPase activity [16]. This high selectivity is important for minimizing off-target effects. In animal models, zastaprazan exhibited dose-dependent inhibition of gastric acid secretion with efficacy comparable to or exceeding other P-CABs in development, and demonstrated significant efficacy in gastric ulcer animal models [16].

A comprehensive phase I clinical trial was conducted in healthy Korean male subjects to evaluate the pharmacokinetics, pharmacodynamics, safety, and tolerability of zastaprazan [17]. This was a randomized, open-label, placebo- and active-controlled study evaluating the effects of single ascending dose and multiple ascending dose regimens of zastaprazan and comparing them with esomeprazole. In a single ascending dose study, healthy subjects received single oral doses of zastaprazan (10–60 mg), esomeprazole (40 mg), or placebo. In the multiple ascending dose study, subjects received once-daily doses of zastaprazan (10, 20, or 40 mg), esomeprazole (40 mg), or placebo for 7 days. Zastaprazan was rapidly absorbed after oral administration, with peak plasma concentrations reached within approximately 0.5 to 1.5 h post-dose in both single and multiple dose studies. The plasma concentration–time profile showed dose-proportional increases in both maximum concentration and area under the curve (AUC) across the studied dose range. A human mass balance study in healthy Chinese male adults who received a single oral dose of 20 mg radiolabeled zastaprazan showed median time to maximum concentration of 0.9 h for both the parent drug and total radioactivity, indicating rapid absorption [18]. Zastaprazan exhibited a plasma mean half-life of approximately 6 to 9 h in the initial study [17], though the mass balance study showed a mean plasma elimination half-life of approximately 28 h for zastaprazan-related material [18], which is considerably longer than PPIs but similar to or longer than other P-CABs. Minimal drug accumulation was noted in the multiple ascending dose study compared with that after once-daily administration for 7 days, suggesting a predictable pharmacokinetic behavior suitable for once-daily dosing. An important difference from some other P-CABs such as vonoprazan and tegoprazan is that zastaprazan demonstrates increased exposure when administered with high-fat meals [19]. This food effect may require consideration in dosing recommendations and patient counseling [15]. The pharmacokinetic properties of zastaprazan compared to other P-CABs and PPIs are summarized in Table 1 [17,20,21,22,23,24,25,26,27].

Zastaprazan undergoes extensive hepatic metabolism. In vitro studies using human hepatocytes identified 18 phase I metabolites and 5 phase II metabolites [28], while the human mass balance study identified 57 metabolites [18]. Based on CYP screening tests and immune-inhibition analysis with CYP antibodies, CYP3A4 and CYP3A5 play major roles in the metabolism of zastaprazan to most of its metabolites. CYP1A2, 2C8, 2C9, 2C19, and 2D6 play minor roles. Uridine diphosphate-glucuronosyltransferase 2B7 and 2B17 are responsible for glucuronidation of certain metabolites. The principal metabolic pathways involve oxidation as phase I reactions and subsequent glucuronidation as phase I/II reactions. The predominance of CYP3A4 and CYP3A5 in zastaprazan metabolism is clinically important because it differs from that of PPIs, which rely mostly on CYP2C19, and suggests that zastaprazan is less likely to show interindividual variability associated with CYP2C19 polymorphisms. This difference in metabolic pathway has important clinical implications for drug–drug interactions. While most PPIs are primarily metabolized by CYP2C19 and function as inhibitors of this enzyme, they can potentially interfere with the metabolism of other CYP2C19 substrates such as clopidogrel, warfarin, diazepam, and phenytoin [29]. In contrast, zastaprazan’s predominant metabolism via CYP3A4 and CYP3A5, rather than CYP2C19, may reduce the potential for clinically significant drug interactions with CYP2C19 substrates, offering a potential advantage in patients requiring multiple medications metabolized through the CYP2C19 pathway. However, this also indicates the potential for drug–drug interactions with CYP3A4 inhibitors or inducers.

The mass balance study showed that within 264 h post-dose, 94.3% of the administered radioactive dose was recovered through excretion, primarily via feces (51.9%) and urine (42.4%) [18]. This indicates that both fecal and urinary excretion contribute substantially to drug elimination. Interestingly, the parent drug remained undetectable in the excreted samples, confirming extensive metabolism before elimination. The blood-to-plasma ratio of total radioactivity, ranging from 0.561 to 0.645, suggests a limited distribution of drug-related material into blood cells.

Unlike the extensive involvement of drug-metabolizing enzymes, drug transporters appear to play a limited role in the disposition of zastaprazan. Studies have shown that zastaprazan and most of its metabolites are not substrates for major drug transporters, including organic cation transporter 1 and 2, organic anion transporter 1 and 3, organic anion transporting polypeptide 1B1 and 1B3, multidrug and toxic compound extrusion 1 and 2 K, P-glycoprotein, and breast cancer-resistant protein [28]. Only the active metabolite M1 showed substrate specificity for P-glycoprotein. This limited transporter involvement suggests that drug–drug interactions mediated by transporters are unlikely to be clinically significant for zastaprazan. An important finding from the phase I study was that pharmacogenomic analysis found no genetic variant of drug-metabolizing enzymes, including CYP2C19, or drug transporters significantly associated with the exposure of zastaprazan [17]. This lack of association with CYP2C19 polymorphisms is a key differentiator from PPIs and suggests a more consistent pharmacokinetic behavior across different patient populations.

**Figure 1:**
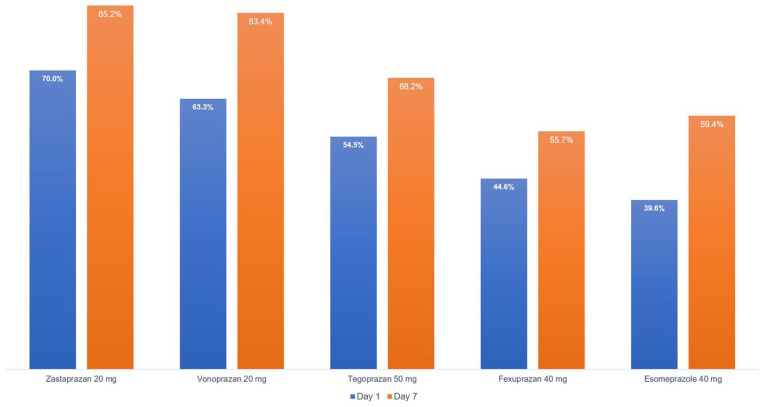
Comparison of mean percentage of time with gastric pH > 4 on day 1 and day 7 among potassium-competitive acid blockers and proton pump inhibitors.

In a phase I study, zastaprazan showed rapid and sustained suppression of gastric acid secretion [17]. The percentage of time that gastric pH > 4 is a key pharmacodynamic parameter for evaluating acid-suppressing agents. After a single dose, the parameter increased in a dose-dependent manner. Notably, zastaprazan 20 mg achieved 70.0% time with pH > 4, and zastaprazan 40 mg achieved 75.0%, both of which were similar to or greater than esomeprazole 40 mg at 41.4% [17]. After 7 days of once-daily administration, the acid suppression effect was maintained, demonstrating favorable pharmacodynamic characteristics for chronic treatment. The sustained effect over multiple days without significant accumulation and the rapid onset of acid suppression, with significant pH elevation observed within 1–2 h of administration, are consistent with the favorable pharmacokinetic profile of zastaprazan. This compares favorably to other P-CABs, with zastaprazan 20 mg achieving 85.2% time with gastric pH > 4 after 7 days, compared to 83.4% with vonoprazan 20 mg, 68.2% with tegoprazan 50 mg, and 55.7% with fexuprazan 40 mg (Figure 1) [17,23,24,30].

Similar to other acid-suppressing agents, zastaprazan administration results in increased serum gastrin levels as a physiological response to profound acid suppression. In a phase I study, serum gastrin levels increased during treatment but returned to near baseline values after discontinuation [17]. A clear exposure-response relationship was observed, with higher systemic exposure to zastaprazan correlating with greater acid suppression. This dose-proportional pharmacodynamic effect supports the rational selection of dosing regimens for various clinical indications.

Given that zastaprazan is primarily metabolized by CYP3A4 and CYP3A5, drug–drug interactions with CYP3A4 inhibitors or inducers are a potential concern. A systematic review and meta-analysis of P-CAB pharmacokinetics found that clarithromycin, a strong CYP3A4 inhibitor, significantly increased the exposure of some P-CABs [19]. Although specific clinical data for zastaprazan-clarithromycin interactions are limited, caution is warranted when co-administering zastaprazan with strong CYP3A4 inhibitors. Conversely, physiologically based pharmacokinetic modeling studies have suggested minimal interaction between zastaprazan and celecoxib [31]. At therapeutic doses, multiple oral doses of zastaprazan did not significantly affect celecoxib exposure. Studies have also indicated minimal interactions between P-CABs including zastaprazan and non-steroidal anti-inflammatory drugs (NSAIDs) or aspirin, supporting safe co-administration for patients requiring gastroprotection while on these medications [19].

## 3. Clinical Studies

To date, two clinical studies on zastaprazan for erosive esophagitis and gastric ulcer have been published (Table 2).

### 3.1. Erosive Esophagitis

A pivotal phase III, multicenter, randomized, double-blind, active-controlled non-inferiority clinical study was conducted to evaluate the efficacy and safety of zastaprazan compared with esomeprazole in patients with erosive esophagitis [32]. The study enrolled 300 Korean subjects with endoscopically confirmed erosive esophagitis according to the LA grades A through D. Subjects were predominantly male with a mean age in the mid-50s and were randomized in a 1:1 ratio to receive either zastaprazan 20 mg once daily or esomeprazole 40 mg once daily for up to 8 weeks. The primary endpoint was the cumulative proportion of subjects with healed erosive esophagitis confirmed by endoscopy at week 8, while secondary endpoints included healing rate at week 4, symptom response including heartburn and acid regurgitation, quality of life assessment using the GERD-health related quality of life (GERD-HRQL) questionnaire, serum gastrin levels, and safety and tolerability.

The majority of patients had mild erosive esophagitis classified as LA grade A/B, which is representative of typical GERD patient populations in the Republic of Korea but limits the ability to assess efficacy in more severe diseases. In the full analysis set, the cumulative healing rates at week 8 were 97.9% and 94.9% for zastaprazan 20 mg and esomeprazole 40 mg, respectively (*p* = 0.178). The study successfully demonstrated the non-inferiority of zastaprazan 20 mg to esomeprazole 40 mg for healing erosive esophagitis at 8 weeks. The 95% confidence interval for the difference in healing rates excluded the pre-specified non-inferiority margin, confirming that zastaprazan was not clinically inferior to esomeprazole. Similar results were observed in the per-protocol set, further supporting the non-inferiority conclusion.

A particularly notable finding was the healing rate at week 4, where zastaprazan 20 mg achieved 95.1% healing compared to 87.7% with esomeprazole 40 mg, representing a statistically significant difference (*p* = 0.026). This statistically significant advantage of zastaprazan in terms of healing speed may be attributed to its rapid and potent acid suppression profile demonstrated in pharmacodynamic studies. Faster healing with zastaprazan could translate to faster symptom relief and improved patient satisfaction. In patients with LA grade C/D, the healing rate in the zastaprazan group was 100% compared with 83.3% in the esomeprazole group; however, due to the limited sample size in the LA grade C/D category, there was no statistically significant difference between the two groups. Both groups showed significant improvements in GERD-related symptoms, including heartburn and acid regurgitation, with a comparable median time to complete resolution of symptoms between the groups. There were no statistically significant differences between the two groups in the proportion of subjects reporting symptom-free days or nights during the treatment period. Improvements in GERD-HRQL scores were observed in both groups, with no significant differences between zastaprazan and esomeprazole, indicating that both treatments effectively improved patients’ quality of life related to their GERD symptoms.

### 3.2. Gastric Ulcers

A phase III, multicenter, randomized, double-blind, active-controlled clinical study was conducted to evaluate the efficacy and safety of zastaprazan compared with lansoprazole in patients with gastric ulcers [33]. The study enrolled 329 Korean subjects with endoscopically confirmed gastric ulcers. Subjects were randomized to receive either zastaprazan 20 mg once daily or lansoprazole 30 mg once daily for up to 8 weeks. The primary endpoint was the cumulative healing rate of gastric ulcers, verified through upper gastrointestinal endoscopy at 8 weeks. The secondary endpoints included the ulcer healing rate at week 4, symptom recovery, quality of life changes, and safety assessment results.

In the per-protocol set, the cumulative healing rate at 8 weeks was remarkably high at 100% for zastaprazan 20 mg and 97.1% for lansoprazole 30 mg (*p* = 0.053). This demonstrated that zastaprazan was non-inferior to lansoprazole in gastric ulcer healing. At week 4, the healing rates were 93.8% for zastaprazan and 91.9% for lansoprazole, showing comparable efficacy at an earlier time point. The excellent healing rates observed with zastaprazan may be attributed to its potent and sustained acid suppression, which maintains gastric pH > 4 for prolonged periods, creating an optimal environment for ulcer healing. The achievement of 100% healing at 8 weeks with zastaprazan represents an outstanding clinical result and suggests that this agent may offer particular advantages in the management of gastric ulcers. Both treatment groups showed improvements in ulcer-related symptoms and quality of life measures.

## 4. Safety

In a phase 3 trial comparing zastaprazan with esomeprazole in patients with erosive esophagitis, the overall safety profile was favorable [32]. The incidence of treatment-emergent adverse events was similar between the two treatment groups, and most events were mild in severity. No serious adverse events were attributed to the study medications, and the types and frequencies of adverse events were consistent with those expected in a GERD patient population. The most frequently reported adverse events in both groups included gastrointestinal disorders such as diarrhea, abdominal discomfort, and nausea, as well as headache, dizziness, and upper respiratory tract infections. These events were generally mild and self-limiting. The rate of treatment discontinuation due to adverse events was low and similar between the groups, indicating the good tolerability of zastaprazan. Similarly, in the gastric ulcer trial comparing zastaprazan with lansoprazole, the incidence of adverse events was comparable between groups, with both treatments demonstrating favorable safety profiles [33].

In a phase 1 study in healthy subjects, zastaprazan was well tolerated at single doses up to 60 mg and multiple doses up to 40 mg once daily for 7 days [17]. No clinically significant changes were observed in vital signs, electrocardiograms, or laboratory parameters, including hematology, clinical chemistry, and urinalysis. Importantly, no signals of hepatotoxicity were detected, which is particularly relevant given that some P-CABs in development have been discontinued because of dose-dependent hepatotoxicity. Specific evaluation of liver injury biomarkers did not reveal any concerning trends. A mass balance study in Chinese subjects also reported a favorable safety profile with no serious adverse events [18]. Nonetheless, given the history of hepatotoxicity with some P-CABs, continued monitoring of liver safety in clinical use is prudent, and post-marketing surveillance and long-term safety studies will be important to fully characterize the hepatic safety profile of zastaprazan.

Gastric acid normally exerts negative feedback on gastrin secretion from G cells in the gastric antrum, and profound acid suppression by PPIs or P-CABs removes this feedback inhibition, leading to compensatory increases in serum gastrin levels. In a phase 3 erosive esophagitis study, serum gastrin levels were measured at baseline and during treatment [32]. Gastrin levels increased in both the zastaprazan and esomeprazole groups during treatment, with mean fold-increases of approximately 2 to 4 fold above baseline for zastaprazan and approximately 2 to 3 fold for esomeprazole. There was a statistically significant difference between the two groups (*p* = 0.047), although both groups showed similar patterns of gastrin elevation. Importantly, gastrin levels in both groups decreased after treatment discontinuation and returned to baseline, indicating the reversibility of hypergastrinemia. In a gastric ulcer trial, gastrin levels similarly increased during treatment with both zastaprazan and lansoprazole and declined after treatment cessation [33]. These findings are consistent with the known effects of acid suppression on gastrin secretion and are similar to observations with other P-CABs and PPIs.

The potential long-term consequences of sustained hypergastrinemia have been extensively studied in the context of PPI therapy. A systematic review by Lundell et al. examined the effects of long-term PPI use, defined as greater than 3 years, on serum gastrin levels and gastric histology [34]. They concluded that long-term PPI use was associated with 1- to 3-fold increases in serum gastrin levels, with significant inter-patient variability, and that enterochromaffin cell hyperplasia was observed, especially in patients infected with *Helicobacter pylori*. However, no evidence of gastric neuroendocrine tumors or adenocarcinomas was observed in these studies of long-term PPI use, including one study with up to 15 years of PPI use. Similar gastrin elevations have been observed with P-CABs. In a study of vonoprazan 20 mg daily compared to lansoprazole 30 mg daily, serum gastrin levels increased 3- to 4-fold with vonoprazan compared to 2- to 3-fold with lansoprazole at 8 weeks [35]. The clinical significance of the slightly higher gastrin elevation with some P-CABs compared to that with PPIs remains to be fully elucidated with long-term follow-up data. For zastaprazan, the gastrin response appears similar to that observed with PPIs, which have a well-established, long-term safety profile. However, continued monitoring of gastrin levels and gastric histology in long-term studies is important to fully characterize the safety of chronic zastaprazan therapy.

Based on its metabolic profile and drug–drug interaction studies, zastaprazan appears to have a manageable interaction profile. Minimal interactions of P-CABs have been demonstrated with NSAIDs and aspirin, and physiologically based pharmacokinetic modeling suggests no significant interaction with celecoxib [19,31]. However, patient counseling and monitoring may be appropriate when co-administering zastaprazan with strong CYP3A4 modulators until more clinical interaction data are available. Data on the use of zastaprazan in special populations are limited. Given that both fecal and urinary excretion contribute to elimination, renal or hepatic impairment could potentially affect the pharmacokinetics of zastaprazan and should be studied. The phase 3 studies included patients up to 75 years of age without apparent safety concerns in older subjects; however, dedicated pharmacokinetic studies in the elderly are needed. No data are available regarding the use of this drug during pregnancy and lactation, and it has not been studied in pediatric populations.

## 5. Clinical Perspectives and Future Development

The clinical development of zastaprazan has demonstrated its efficacy in acid-related disorders. With its approval in the Republic of Korea for erosive GERD and gastric ulcer, zastaprazan represents an important alternative to PPIs, particularly for patients requiring rapid symptom relief and mucosal healing. The achievement of 100% healing rate in gastric ulcers at 8 weeks and significantly faster healing of erosive esophagitis at week 4 compared to standard PPI therapy suggests particular advantages for these indications. The ability to achieve these outcomes with a relatively low dose of 20 mg once daily, compared to some other P-CABs that require 40–50 mg dosing, indicates high potency and favorable pharmacodynamic properties. While zastaprazan metabolism is not significantly affected by CYP2C19 polymorphisms, providing an advantage over PPIs in terms of consistent efficacy across patient populations, the food effect on exposure warrants consideration in clinical use, although consistent dosing relative to meals may mitigate this concern.

Zastaprazan is undergoing active clinical development for additional indications, with phase 3 studies evaluating its use for the prevention of peptic ulcers in patients requiring chronic NSAID therapy and for the treatment of non-erosive reflux disease (NERD). This represents a significant unmet need, as NSAID-induced gastropathy remains a common clinical problem, and P-CABs may offer advantages through more consistent and potent acid suppression compared to PPIs [36]. Based on the established uses of other P-CABs and PPIs, potential future development areas for zastaprazan could include NERD, as a large proportion of GERD patients have non-erosive disease, and demonstrating efficacy in this population would significantly expand the eligible patient base. *H. pylori* eradication is another potential area, as P-CABs have shown promise as part of dual or triple therapy regimens, potentially offering higher cure rates than PPI-based regimens because of more potent acid suppression [37,38]. The ability to achieve higher intragastric pH enhances the efficacy of pH-dependent antibiotics. Long-term maintenance therapy to prevent GERD recurrence is commonly needed, and studies evaluating the optimal maintenance doses and long-term safety and efficacy of zastaprazan are important. Other potential applications include acid suppression to prevent rebleeding in patients with upper gastrointestinal hemorrhage, where the rapid onset of action of zastaprazan could be advantageous.

Several important questions regarding zastaprazan remain. The phase 3 erosive esophagitis study predominantly enrolled LA grade A/B patients, and efficacy in LA grade C/D patients requires further documentation. Whether zastaprazan provides superior efficacy in patients who have failed PPI therapy remains a key question. The efficacy and optimal dosing for non-erosive GERD need to be established, as do the optimal dose and safety profile for long-term maintenance therapy beyond 6–12 months. Head-to-head trials comparing zastaprazan with vonoprazan, fexuprazan, or tegoprazan would help clarify the relative advantages within the P-CAB class. As a newer agent, cost-effectiveness studies comparing zastaprazan with PPIs and other P-CABs are needed. Data from routine clinical practice will be important for confirming the efficacy and safety observed in clinical trials. Further clarification on the clinical impact of the food effect and optimal dosing instructions relative to meals is needed, and safety and efficacy in elderly, renally impaired, hepatically impaired, pregnant, and pediatric populations require further study.

The potential role of zastaprazan in pediatric populations warrants consideration, particularly given the recognized limitations of PPI therapy in children. Recent systematic reviews have demonstrated that while PPIs effectively treat erosive esophagitis and achieve symptom control in older children and adolescents, they show limited efficacy in infants with GERD, performing no better than placebo in this age group [39]. This therapeutic gap, combined with concerns about long-term PPI use in developing children, suggests a possible niche for alternative acid suppression strategies. Zastaprazan’s pharmacological profile—including rapid onset, consistent acid suppression independent of CYP2C19 polymorphisms, and once-daily dosing—could theoretically address several challenges encountered with pediatric PPI therapy. However, before zastaprazan can be considered for pediatric use, rigorous age-appropriate clinical development is essential. This includes pharmacokinetic studies across pediatric age groups to establish appropriate dosing, given developmental changes in drug metabolism and clearance. Safety evaluation must address both short-term tolerability and potential long-term effects on growth, bone mineral density, nutrient absorption, and gut microbiome development—concerns already recognized with pediatric PPI use. Regulatory pathways for pediatric drug development require dedicated clinical trials with endpoints validated for children, informed consent procedures appropriate for minors, and careful risk-benefit assessment at each developmental stage. Until such studies are completed and reviewed by regulatory authorities, zastaprazan use should remain restricted to approved adult indications, with pediatric application considered only within the context of properly designed clinical trials.

While currently approved only in the Republic of Korea, regulatory submissions for zastaprazan in other countries are anticipated in the future. Successful registration in other Asian markets, North America, and Europe would significantly expand its clinical impact. International clinical trials and real-world evidence studies will be important for supporting these regulatory efforts. Standard monitoring, as with other acid suppressants, is appropriate, with consideration for periodic assessment of serum gastrin levels with long-term use and monitoring for potential drug interactions if co-administered with strong CYP3A4 modulators. To fully establish the role of zastaprazan in managing acid-related disorders, clinical trials should evaluate its efficacy in PPI-refractory disease, NERD, long-term maintenance therapy, severe erosive esophagitis, and *H. pylori* eradication combination therapy. Long-term studies should examine the effects of hypergastrinemia on gastric histology, effects on gastric microbiome, and potential effects on nutrient absorption. Real-world evidence from post-marketing surveillance studies and large database analyses of effectiveness and safety are also needed.

## 6. Conclusions

Zastaprazan is a newly developed P-CAB that has demonstrated favorable pharmacological properties and clinical efficacy for the treatment of acid-related disorders. The drug is characterized by potent and selective H^+^/K^+^-ATPase inhibition, rapid absorption with onset of acid suppression within 1–2 h, and a moderate to long half-life allowing once-daily dosing with sustained 24 h acid control. In erosive esophagitis, zastaprazan 20 mg showed non-inferiority to esomeprazole 40 mg at week 8, and importantly demonstrated superior healing at week 4. In gastric ulcers, zastaprazan 20 mg also showed non-inferiority to lansoprazole 30 mg at 8 weeks. The drug is effective at a relatively low dose of 20 mg once daily and provides potent acid suppression, with 85.2% time with pH > 4 after 7 days, which is superior to lansoprazole and comparable to or exceeding other P-CABs.

The safety profile of zastaprazan is favorable, with adverse event profiles similar to those of PPIs across clinical trials, no hepatotoxicity signals observed, and reversible increases in serum gastrin levels, similar to PPIs and other P-CABs. Zastaprazan is approved in the Republic of Korea for treatment of erosive esophagitis and gastric ulcer, and represents an important addition to the therapeutic armamentarium for acid-related disorders, offering clinicians and patients an effective alternative to traditional PPIs with potential advantages in rapid onset, sustained acid suppression, and consistent efficacy across patient populations. Further studies are needed to establish its role in PPI-refractory disease, non-erosive GERD, severe erosive esophagitis, and long-term maintenance therapy, as well as to define its position relative to other P-CABs through head-to-head comparisons. Pediatric application warrants careful investigation through appropriately designed clinical trials addressing developmental pharmacokinetics, safety, and efficacy across age groups. As clinical experience accumulates through post-marketing surveillance and real-world use, the full therapeutic potential of zastaprazan in managing the spectrum of acid-related disorders will become more evident.

## Figures and Tables

**Table 1:** Pharmacokinetic properties of zastaprazan compared to other P-CABs and PPIs.

Drug	Structure	MW	pKa	C_max_ (μg/L)	T_max_ (h)	AUC (μg·h/L)	Half-Life (h)
Zastaprazan 20 mg	Imidazopyridine	554.59	9.9	357.54 ± 109.63	0.8 (0.5–1.5)	1166.8 ± 362.1	7.7 ± 1.8
Vonoprazan 20 mg	Sulfonyl pyrrole	345.39	9.1	25.0 ± 5.6	1.5 (1.5–3.0)	160.3 ± 38.6	6.9 ± 0.8
Tegoprazan 50 mg	Benzimidazole	387.38	5.1	669.9 ± 30.3	1.0 (0.5–2.0)	2519.8 ± 8.7	3.7 ± 0.5
Fexuprazan 40 mg	Sulfonyl pyrrole	410.41	8.4	40.9 ± 17.3	2.0 (1.5–4.0)	439.8 ± 163.1	9.1 ± 1.2
Lansoprazole 30 mg	Benzimidazole	369.37	3.8	600–1200	1.5–3.3	1700–5000	2.0–4.2
Rabeprazole 20 mg	Benzimidazole	359.44	4.5	410	2.6–3.4	800	1.8–2.4
Esomeprazole 40 mg	Benzimidazole	345.42	4.1	5130	1.0–3.5	7300	0.8

MW, molecular weight; C_max_, maximum plasma concentration; T_max_, time to reach C_max_; AUC, area under the plasma concentration–time curve.

**Table 2:** Clinical efficacy of zastaprazan in acid-related disorders.

Disorders	Subject No.	Treatment	Healing Rate							
			Full Analysis Set				Per-Protocol Set			
			Week 4	*p*-Value	Week 8	*p*-Value	Week 4	*p*-Value	Week 8	*p*-Value
Erosive esophagitis [32]	300	Zastaprazan 20 mg vs. esomeprazole 40 mg	95.1% vs. 87.7%	0.026	97.9% vs. 94.9%	0.178	97.1% vs. 92.6%	0.101	100% vs. 97.5%	0.063
Gastric ulcer [33]	329	Zastaprazan 20 mg vs. lansoprazole 30 mg	88.3% vs. 89.8%	0.718	94.4% vs. 94.7%	1.000	93.8% vs. 91.9%	0.644	100% vs. 97.1%	0.053

## Data Availability

No new data were created or analyzed in this study.

## Abbreviations

The following abbreviations are used in this manuscript:

AUC	area under the curve
CYP	cytochrome P450
GERD	gastroesophageal reflux disease
GERD-HRQL	GERD-health related quality of life
H_2_RA	histamine-2-receptor antagonist
LA	Los Angeles
NERD	non-erosive reflux disease
NSAID	non-steroidal anti-inflammatory drug
P-CAB	potassium-competitive acid blocker
PPI	proton pump inhibitor
PUD	peptic ulcer disease

## References

[1] Katz P.O., Dunbar K.B., Schnoll-Sussman F.H., Greer K.B., Yadlapati R., Spechler S.J. (2022). ACG Clinical Guideline for the Diagnosis and Management of Gastroesophageal Reflux Disease. Am. J. Gastroenterol..

[2] Eusebi L.H., Ratnakumaran R., Yuan Y., Solaymani-Dodaran M., Bazzoli F., Ford A.C. (2018). Global prevalence of, and risk factors for, gastro-oesophageal reflux symptoms: A meta-analysis. Gut.

[3] Xie X., Ren K., Zhou Z., Dang C., Zhang H. (2022). The global, regional and national burden of peptic ulcer disease from 1990 to 2019: A population-based study. BMC Gastroenterol..

[4] Wang W.H., Huang J.Q., Zheng G.F., Xia H.H., Wong W.M., Lam S.K., Wong B.C. (2005). Head-to-head comparison of H2-receptor antagonists and proton pump inhibitors in the treatment of erosive esophagitis: A meta-analysis. World J. Gastroenterol..

[5] Li M.J., Li Q., Sun M., Liu L.Q. (2017). Comparative effectiveness and acceptability of the FDA-licensed proton pump inhibitors for erosive esophagitis: A PRISMA-compliant network meta-analysis. Medicine.

[6] Huh C.W., Chang J.W., Son N.H., Jung D.H., Jung H.K., Kang S.J., Kim S.Y., Choi M., Jeong D.M., Kim H.J. (2026). 2025 Focused Update of the Seoul Consensus on Gastroesophageal Reflux Disease: Evidence-based Recommendations on Acid Suppressive Therapy. J. Neurogastroenterol. Motil..

[7] Savarino V., Dulbecco P., de Bortoli N., Ottonello A., Savarino E. (2017). The appropriate use of proton pump inhibitors (PPIs): Need for a reappraisal. Eur. J. Intern. Med..

[8] Harris D.M., Stancampiano F.F., Burton M.C., Moyer A.M., Schuh M.J., Valery J.R., Bi Y. (2021). Use of Pharmacogenomics to Guide Proton Pump Inhibitor Therapy in Clinical Practice. Dig. Dis. Sci..

[9] Kim G.H., Fass R. (2025). Potassium-competitive Acid Blockers for Treatment of Extraesophageal Symptoms and Signs. J. Neurogastroenterol. Motil..

[10] Gralnek I.M., Dulai G.S., Fennerty M.B., Spiegel B.M. (2006). Esomeprazole versus other proton pump inhibitors in erosive esophagitis: A meta-analysis of randomized clinical trials. Clin. Gastroenterol. Hepatol..

[11] Zerbib F., Bredenoord A.J., Fass R., Kahrilas P.J., Roman S., Savarino E., Sifrim D., Vaezi M., Yadlapati R., Gyawali C.P. (2021). ESNM/ANMS consensus paper: Diagnosis and management of refractory gastro-esophageal reflux disease. Neurogastroenterol. Motil..

[12] Hunt R.H., Scarpignato C. (2015). Potassium-Competitive Acid Blockers (P-CABs): Are They Finally Ready for Prime Time in Acid-Related Disease?. Clin. Transl. Gastroenterol..

[13] Seo S., Jung H.K., Gyawali C.P., Lee H.A., Lim H.S., Jeong E.S., Kim S.E., Moon C.M. (2024). Treatment Response with Potassium-competitive Acid Blockers Based on Clinical Phenotypes of Gastroesophageal Reflux Disease: A Systematic Literature Review and Meta-analysis. J. Neurogastroenterol. Motil..

[14] Abdel-Aziz Y., Metz D.C., Howden C.W. (2021). Review article: Potassium-competitive acid blockers for the treatment of acid-related disorders. Aliment. Pharmacol. Ther..

[15] Blair H.A. (2024). Zastaprazan: First Approval. Drugs.

[16] Ku J.M., Cho J.H., Kim K., Kim J.Y., Kim J.Y., Kim J., Cha H., Cheon B. (2023). JP-1366: A novel and potent potassium-competitive acid blocker that is effective in the treatment of acid-related diseases. Pharmacol. Res. Perspect..

[17] Hwang I., Ji S.C., Oh J., Kim H., Cha H., Kim J., Lee C.S., Yu K.S., Lee S. (2023). Randomised clinical trial: Safety, tolerability, pharmacodynamics and pharmacokinetics of zastaprazan (JP-1366), a novel potassium-competitive acid blocker, in healthy subjects. Aliment. Pharmacol. Ther..

[18] Liu X., Deng D., Meng J., Hu H., Zhuang Q., Zhou X., Fu L., Fan B., Xu X., Huang Q. (2025). Pharmacokinetics, mass balance, and metabolism of the novel potassium-competitive acid blocker JP-1366 in healthy Chinese adults following a single oral dose of [(14)C]JP-1366. Front. Pharmacol..

[19] Liu J., Hahn J. (2025). Clinical pharmacokinetics of potassium competitive acid blockers: A systematic review and meta-analysis. Front. Pharmacol..

[20] Scarpignato C., Hunt R.H. (2024). Potassium-competitive Acid Blockers: Current Clinical Use and Future Developments. Curr. Gastroenterol. Rep..

[21] Ramani A., Merchant A., Cash B.D. (2023). Review of the clinical development of fexuprazan for gastroesophageal reflux-related disease. Eur. J. Clin. Pharmacol..

[22] Yang E., Hwang I., Ji S.C., Kim J., Lee S. (2024). Population pharmacokinetic analysis of zastaprazan (JP-1366), a novel potassium-competitive acid blocker, in patients and healthy volunteers. CPT Pharmacomet. Syst. Pharmacol..

[23] Sunwoo J., Oh J., Moon S.J., Ji S.C., Lee S.H., Yu K.S., Kim H.S., Lee A., Jang I.J. (2018). Safety, tolerability, pharmacodynamics and pharmacokinetics of DWP14012, a novel potassium-competitive acid blocker, in healthy male subjects. Aliment. Pharmacol. Ther..

[24] Jenkins H., Sakurai Y., Nishimura A., Okamoto H., Hibberd M., Jenkins R., Yoneyama T., Ashida K., Ogama Y., Warrington S. (2015). Randomised clinical trial: Safety, tolerability, pharmacokinetics and pharmacodynamics of repeated doses of TAK-438 (vonoprazan), a novel potassium-competitive acid blocker, in healthy male subjects. Aliment. Pharmacol. Ther..

[25] Yang Z.C., Yu F., Wang Y.Q., Wei J.F. (2016). Stereoselective Pharmacodynamics and Pharmacokinetics of Proton Pump Inhibitors. Curr. Drug Metab..

[26] Shin J.M., Sachs G. (2008). Pharmacology of proton pump inhibitors. Curr. Gastroenterol. Rep..

[27] Sachs G., Shin J.M., Howden C.W. (2006). Review article: The clinical pharmacology of proton pump inhibitors. Aliment. Pharmacol. Ther..

[28] Lee M.S., Lee J., Pang M., Kim J., Cha H., Cheon B., Choi M.K., Song I.S., Lee H.S. (2024). In Vitro Metabolism and Transport Characteristics of Zastaprazan. Pharmaceutics.

[29] Zvyaga T., Chang S.Y., Chen C., Yang Z., Vuppugalla R., Hurley J., Thorndike D., Wagner A., Chimalakonda A., Rodrigues A.D. (2012). Evaluation of six proton pump inhibitors as inhibitors of various human cytochromes P450: Focus on cytochrome P450 2C19. Drug Metab. Dispos..

[30] Sunwoo J., Ji S.C., Oh J., Ban M.S., Nam J.Y., Kim B., Song G.S., Yu K.S., Jang I.J., Lee S. (2020). Pharmacodynamics of tegoprazan and revaprazan after single and multiple oral doses in healthy subjects. Aliment. Pharmacol. Ther..

[31] Choi S.C., Kim J., Lim H.S. (2025). Evaluation of drug-drug interaction potentials between JP-1366 and celecoxib using physiologically based pharmacokinetic modeling. Transl. Clin. Pharmacol..

[32] Oh J.H., Kim H.S., Cheung D.Y., Lee H.L., Lee D.H., Kim G.H., Choi S.C., Cho Y.K., Chung W.C., Kim J.W. (2025). Randomized, Double-Blind, Active-Controlled Phase 3 Study to Evaluate Efficacy and Safety of Zastaprazan Compared With Esomeprazole in Erosive Esophagitis. Am. J. Gastroenterol..

[33] Park K.S., Kim H.S., Oh J.H., Chung W.C., Choi S.C., Lee S.H., Kim T.H., Cheung D.Y., Baik G.H., Kim S.M. (2026). Randomized, Double-Blind, Active-Controlled, Parallel, Phase 3 Clinical Trial for Evaluating the Efficacy and Safety of Zastaprazan in Patients with Gastric Ulcers. Gut Liver.

[34] Lundell L., Vieth M., Gibson F., Nagy P., Kahrilas P.J. (2015). Systematic review: The effects of long-term proton pump inhibitor use on serum gastrin levels and gastric histology. Aliment. Pharmacol. Ther..

[35] Ashida K., Sakurai Y., Hori T., Kudou K., Nishimura A., Hiramatsu N., Umegaki E., Iwakiri K. (2016). Randomised clinical trial: Vonoprazan, a novel potassium-competitive acid blocker, vs. lansoprazole for the healing of erosive oesophagitis. Aliment. Pharmacol. Ther..

[36] Kim G.H., Choi M.G., Kim J.I., Lee S.T., Chun H.J., Lee K.L., Choi S.C., Jang J.Y., Lee Y.C., Kim J.G. (2023). Efficacy and Safety of Fexuprazan in Patients with Acute or Chronic Gastritis. Gut Liver.

[37] Ma Z., Wang S., Wang F., Ren Q. (2025). Research progress of potassium-competitive acid blockers in the treatment of Helicobacter pylori. Ann. Med..

[38] Jung Y.S., Kim S., Kim H.Y., Noh S.J., Park J.H., Sohn C.I., Park C.H. (2023). Efficacy and Tolerability of 14-Day Tegoprazan- versus Rabeprazole-Based Triple Therapy for Eradication of Helicobacter pylori: A Real-World Evidence Study. Gut Liver.

[39] Fernandez-Gonzalez S.M., Moreno-Alvarez A., Solar-Boga A. (2024). Proton Pump Inhibitors in Pediatric Gastroesophageal Reflux Disease: A Systematic Review of Randomized Controlled Trials. Children.

